# Evening home pulse pressure predicted cardiovascular events and mortality in older adults with hypertension: findings based on the STEP trial

**DOI:** 10.1038/s41440-025-02349-y

**Published:** 2025-08-26

**Authors:** Yufei Ji, Xinyi Peng, Sifei Chen, Qirui Song, Jingjing Bai, Jun Cai

**Affiliations:** 1 Hypertension Center, Fuwai Hospital, State Key Laboratory of Cardiovascular Disease, National Center for Cardiovascular Diseases, Peking Union Medical College, Chinese Academy of Medical Sciences, Beijing, China; 2 Beijing Anzhen Hospital, Capital Medical University, Beijing Institute of Heart, Lung and Blood Vessel Diseases, Beijing, China; 3 Hypertension Center, Fuwai Hospital, Peking Union Medical College, Chinese Academy of Medical Sciences, Beijing, China

**Keywords:** Pulse Pressure, Home Blood Pressure Monitoring, Hypertension, Cardiovascular disease, Risk Prediction

## Abstract

Hypertension is a key predictor of cardiovascular disease (CVD) and mortality. Home blood pressure monitoring (HBPM) is a cost-effective way to assess CVD risk, though existing research mainly focuses on morning systolic (SBP) and diastolic (DBP) blood pressure measurements. This study aimed to evaluate whether evening pulse pressure (PP) measured at home could better predict CVD risk and mortality in Chinese older adults with hypertension. Data from the STEP trial, a multicenter, randomized controlled trial, were analyzed. Morning and evening home BP was measured twice a day at least once a week from enrollment to 12 months of follow-up, based on which PP was calculated and categorized into tertiles. The primary outcome was a composite of cardiovascular events and all-cause mortality. Among 7703 participants included in this analysis, 284 composite events occurred during a median follow-up of 3.43 years. Compared to the first tertile evening PP group, the third tertile evening PP group exhibited a 60% (HR = 1.60; 95% CI: 1.12–2.29) higher risk of primary outcome in the final adjusted model. A 26% increased risk was observed with each tertile increment (HR = 1.26; 95% CI: 1.05–1.50, *P*_trend_ = 0.0112). For each 10 mmHg increase in evening PP, the risk of outcome events increased by approximately 33%. Higher evening home PP significantly predicts an increased risk of composite events (NRI: 0.16, 95% CI: 0.02–0.29), unlike morning SBP, PP, or evening SBP. Efforts to monitor evening home PP may be an effective strategy to improve BP control and prevent CVD and mortality. **Trial Registration:** STEP ClinicalTrials.gov number, NCT03015311.

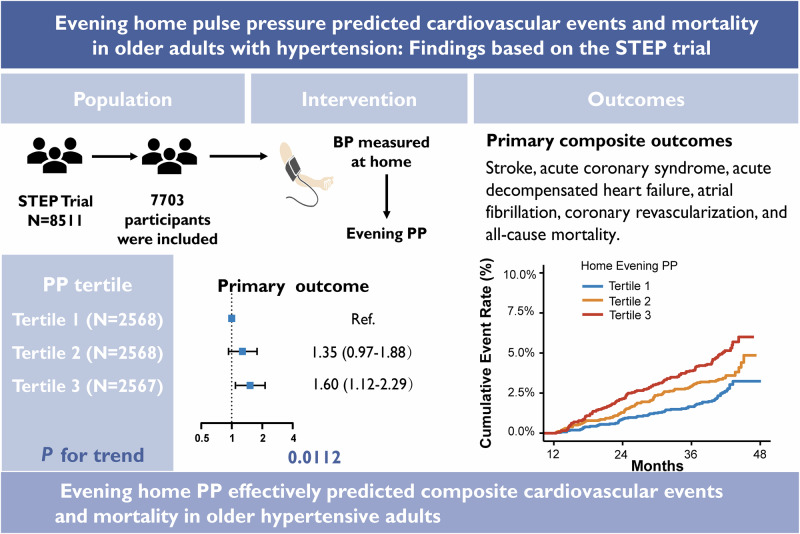

## Introduction

Hypertension, the strongest cardiovascular disease (CVD) predictor, is a significant global health concern associated with high morbidity and mortality [1, 2]. Its disease burden is exceptionally high in Asian countries experiencing the double burden of population aging and hypertension. One-fifth of the Chinese population is aged ≥60, and over half of them have hypertension [3, 4]. Therefore, it is paramount to monitor and control blood pressure (BP) to prevent further CVD and related adverse outcomes among this population.

Home blood pressure monitoring (HBPM) is a reliable, convenient, and cost-effective approach to the monitoring and management of hypertension. Compared to conventional office BP monitoring, HBPM enhances treatment adherence and BP control through easy availability, higher patient involvement, timely feedback, better reproducibility, lower costs, and avoidance of white-coat effect [5–7]. Growing evidence has demonstrated the predictive effects of HBPM on CVD risk and mortality [7, 8]. International guidelines have also highlighted the importance of HBPM, recommending the measurement to be performed both in the morning and evening for comprehensive assessment of BP fluctuations and better BP management [9–11].

Despite the benefits and recommendations of HBPM in BP management, the best measurement time remains debated. A large body of evidence has supported the predicting performance of morning home BP in subsequent CVD risk and mortality. For instance, the J-HOP study [12] and the Ohasama Study [13], both based on the Japanese population, have shown that morning HBPM better predicts stroke risk than evening HBPM. However, there are also a few studies showing equal predictive abilities between morning and evening BP. For instance, the Finn-Home Study [14] in Finland has found that morning and evening HBPM are equally predictive of fatal and nonfatal CVD. This discrepancy may be explained by the different populations, indicators, and outcomes. The relatively few studies focusing on evening HBPM have indicated the need for further evidence on its predicting effectiveness. Compared to morning HBPM, evening HBPM has less stringent measurement requirements [15]. Taken before bedtime, evening readings enjoy a more flexible schedule, potentially enhancing adherence and yielding superior outcomes [16].

Additionally, most previous HBPM studies are based on systolic blood pressure (SBP) and diastolic blood pressure (DBP), the already known definite predictors of CVD and mortality [8]. Few studies have focused on pulse pressure (PP), the numerical difference between SBP and DBP, though increasing evidence suggests it as a potential predictor of CVD and mortality [17–19]. To fill the research gap, this study investigated the predictive validity of evening PP measured at home in determining the risk of CVD and mortality and compared it with morning home PP using a nationwide Chinese database of older adults with hypertension.

Point of view

- Clinical relevance: Evening home PP effectively predicts cardiovascular events and mortality in older adults with hypertension.
- Future direction: Future research should focus on standardizing evening home PP measurement, determining its optimal threshold, conducting long-term studies, and validating its applicability across diverse populations to further advance its clinical implementation.
- Consideration for the Asian population: This study specifically investigates older adults with hypertension in China; however, extrapolation to other populations requires careful consideration of cultural, genetic, and socioeconomic differences to ensure intervention accessibility and efficacy.

## Methods

### Study design and participants

The current study was derived from the Strategy of BP Intervention in the Elderly Hypertensive Patients (STEP) trial, and the details of the STEP trial’s rationality, design, and procedures have been published elsewhere [20, 21]. The STEP trial was a multicenter, randomized controlled trial to examine the effectiveness of different BP control targets in preventing CVD among people aged 60 to 80 years [20]. Briefly, 8511 older adults with primary hypertension were recruited at 42 clinical centers throughout China from January 2017 and followed up until December 2020, with a median follow-up of 3.35 years. Older Chinese adults aged 60 to 80 years were included if they had hypertension with SBP of 140 to 190 mmHg or were on antihypertensive medication. Participants were excluded if they had a history of ischemic or hemorrhagic stroke. The participants were randomly assigned to either an intensive (110 ≤ SBP target < 130 mmHg) or a mild (130 ≤ SBP target < 150 mmHg) treatment group to compare various clinical outcomes. The STEP study protocol was approved by the institutional review board of Fuwai Hospital and each participating institution, and written informed consent was obtained from all participants.

### Home BP measurement

Home BP was measured twice in the morning before medication and twice in the evening before sleep at least one day per week during follow-up. Each patient was provided with a validated Omron HEM-9200T automatic (Omron Healthcare, Kyoto, Japan) to measure their BP at home [22]. The morning BP was measured during the period of 6:00 a.m. to 9:00 a.m., while the evening BP was measured between 6:00p.m. and 9:00 p.m., and the BP data were automatically uploaded to the data collection center [22]. Moreover, participants were required to rest for at least five minutes in a seated position before the initial BP measurement, and BP was measured twice at least one minute apart. A third BP measurement was performed if the first two readings differed more than 5 mmHg. The mean of all available measurements was used for analysis.

To conduct the current analysis, the average morning and evening home SBP, DBP, and PP were computed for each patient. These values were derived from the mean of the patient’s BP measurements taken from enrollment to 12 months after enrollment. The morning and evening home PP was obtained by calculating the difference between the average morning and evening home SBP and DBP readings, based on which participants were divided into three tertile groups.

### Study outcomes

The primary outcome was the occurrence of stroke (ischemic or hemorrhagic), acute coronary syndrome (acute myocardial infarction or hospitalization for unstable angina), acute decompensated heart failure, atrial fibrillation, coronary revascularization, and all-cause mortality. The secondary outcomes were stroke, acute coronary syndrome, major cardiovascular events (MACE), and all-cause mortality. MACE was defined as a composite of the first occurrence of acute coronary syndrome, acute decompensated heart failure, coronary revascularization, and cardiovascular mortality. The first event was included in the analysis if two or more events occurred in the same patient. The definition of the outcomes in this study was consistent with the definition in the STEP trial. All clinical outcomes were finally assessed by the STEP event adjudication committee.

### Statistical analysis

Participants were divided into three groups based on the tertiles of evening home PP. Baseline characteristics were summarized as means ± SDs for continuous variables and numbers and proportions for categorical variables. We compared the differences in baseline characteristics among the three PP groups using one-way ANOVA for continuous data and the Pearson *χ*^2^ test for categorical variables. Similar baseline analyses were also performed for morning SBP, PP, and evening SBP.

Cumulative event rates were estimated for outcome for the three evening PP groups using the Kaplan–Meier method, and differences in survival distributions were assessed using the log-rank test. Cox proportional regression models were used to evaluate the associations between evening PP (first as categorical variables, then as continuous variables) and the outcomes with two models. Model 1 (partially adjusted model) adjusted for treatment group, sex, age, and study site. Model 2 (fully adjusted model) further adjusted for BMI, evening home SBP at baseline, heart rate, glucose level, smoking, alcohol use, and coronary heart disease history. Multicollinearity was assessed using the variance inflation factor (VIF) [23]. We also assessed the primary outcome across the evening home PP (as a continuous variable) by using cubic spline regression model, adjusting for the aforementioned covariates. The cubic spline function was fitted using three knots.

To rule out the possibility of reverse causality, we tested the associations between PP and outcomes after excluding participants with a history of coronary heart disease. As part of the sensitivity analysis, we also examined the association between morning SBP, PP, evening SBP, and the primary outcome. In addition, we calculated the net reclassification improvement (NRI) to examine the incremental prognostic value of PP compared with the traditional model for predicting the risk of composite events [24]. A significantly positive NRI indicates improved performance of a new risk prediction model after adding new variables as compared to the existing model [24]. All analyses were performed using R Version 4.3.1 (R Foundation for Statistical Computing, Vienna, Austria). Two-sided *P* < 0.05 was considered statistically significant.

## Results

### Sample characteristics

Among the 8511 participants included in the STEP trial, 808 were excluded due to incomplete data on evening mean SBP and DBP, withdrawing from the trial, or experiencing the primary outcome during the 12 months of follow-up, leading to 7703 participants included in the final analysis (Supplementary Fig. 1). Their mean age was 66.12 ± 4.78, and 46.7% were male. The number of participants in the first, second, and third evening PP tertiles was 2568, 2568, and 2567, respectively (Table 1). Participants in the higher evening PP tertiles tended to be older and had a higher prevalence of coronary heart disease (Table 1).

**Table 1:** Baseline participant characteristics across evening home pulse pressure tertiles

Variables	Overall (*N* = 7703)	Tertile 1 (*N* = 2568)	Tertile 2(*N* = 2568)	Tertile 3(*N* = 2567)
Age, *y*	66.12 (4.78)	64.66 (4.19)	66.08 (4.66)	67.62 (4.98)
Male, *n* (%)	3594 (46.7)	1325 (51.6)	1151 (44.8)	1118 (43.6)
BMI, kg/m^2^	25.59 (3.15)	25.52 (3.05)	25.57 (3.10)	25.66 (3.30)
Evening PP during follow-up, mmHg	51.49 (9.08)	42.04 (3.91)	50.90 (2.11)	61.53 (6.08)
Home blood pressure at baseline				
Evening SBP, mmHg	129.44 (11.34)	122.99 (9.12)	129.12 (9.39)	136.20 (11.28)
Evening DBP, mmHg	78.58 (7.93)	81.42 (7.13)	78.91 (7.33)	75.42 (8.12)
Evening PP, mmHg	50.85 (10.11)	41.57 (5.40)	50.22 (5.06)	60.78 (8.19)
Evening HR, bpm	73.57 (10.34)	74.90 (10.18)	73.36 (10.27)	72.44 (10.42)
Smoking, *n* (%)				
Currently	1251 (16.2)	431 (16.8)	391 (15.2)	429 (16.7)
Never	5520 (71.7)	1791(69.7)	1902 (74.1)	1827 (71.2)
Former	932 (12.1)	346 (13.5)	275 (10.7)	311 (12.1)
Alcohol use, *n* (%)				
Currently	2033 (26.4)	784 (30.5)	632 (24.6)	617 (24.0)
Never	5278 (68.5)	1657 (64.5)	1811 (70.5)	1810 (70.5)
Former	392 (5.1)	127 (4.9)	125 (4.9)	140 (5.5)
Lipid profile				
Triglyceride, mmol/L	1.60 (1.07)	1.58 (1.08)	1.59 (1.02)	1.61 (1.12)
TC, mmol/L	4.89 (1.09)	4.89 (1.10)	4.88 (1.08)	4.91 (1.09)
HDL-C, mmol/L	1.26 (0.31)	1.27 (0.31)	1.26 (0.30)	1.26 (0.31)
LDL-C, mmol/L	2.70 (0.88)	2.69 (0.87)	2.69 (0.88)	2.71 (0.89)
FBG, mmol/L	6.13 (1.56)	5.90 (1.41)	6.11 (1.51)	6.38 (1.70)
Creatinine, μmol/L	73.10(17.88)	73.70 (17.37)	72.27 (17.66)	73.34 (18.60)
With DM history, *n* (%)	1470 (19.1)	302 (11.8)	480 (18.7)	688 (26.8)
With CHD history, *n* (%)	453 (5.9)	121 (4.7)	165 (6.4)	167 (6.5)

Values are presented as the mean ± SD or *n* (%), as appropriate

*BMI* body mass index, *SBP* systolic blood pressure, *DBP* diastolic blood pressure, *HR* heart rate, *TC* total cholesterol, *HDL-C* high-density lipoprotein cholesterol, *LDL-C* low-density lipoprotein cholesterol, *FBG* fasting blood glucose, DM diabetes mellitus, *CHD* coronary heart disease

### Association between evening home PP and outcome events

**Fig. 1:**
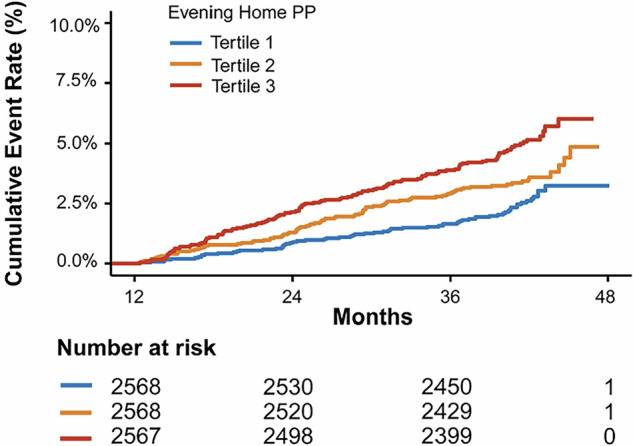
Kaplan–Meier curves for evening home pulse pressure and primary outcome. The highest tertile group had a significantly lower incidence of cardiovascular disease compared to the lowest tertile group, as assessed by the log-rank test (*P* < 0.0001)

During a median follow-up of 3.43 years, there were a total of 284 outcome events. For evening home PP, the Kaplan–Meier survival function curves showed a higher cumulative incidence of primary outcome in the highest tertile group than in the lowest tertile group (log-rank test *P* < 0.0001, Fig. 1). Table 2 presents the association between evening PP and the primary outcome, with adjusting for covariates. The VIF ranges from 1.0 to 1.8, below the threshold of 5 for multicollinearity. Compared to the first PP tertile group, the second PP tertile group had a significantly higher risk of composite events in model 1(HR = 1.40, 95% CI:1.01–1.93), and the third PP tertile group had a significantly higher risk of composite events in both model 1 (HR = 1.76, 95% CI: 1.28–2.41) and model 2 (HR = 1.60, 95% CI: 1.12–2.29). Furthermore, a significant increase in the risk of composite events was observed with each tertile increment in model 1 (HR = 1.32; 95% CI: 1.13–1.54, *P*_trend_ = 0.0004), and model 2 (HR = 1.26; 95% CI: 1.05–1.50, *P*_trend_ = 0.0112) (Table 2).

**Table 2:** Adjusted association of evening home pulse pressure (subgroups or per 10 mmHg increase) and primary outcome

	Overall	Tertile 1 (*N* = 2568)	Tertile 2(*N* = 2568)	Tertile 3 (*N* = 2567)	Per one stage increase	*P*_trend_	Per 10 mmHg increase	*P*_value_
Outcomes (*n*)	284	65	92	127	—	—	—	---
IR*(95% CI)	11.02 (9.79–12.39)	7.52 (5.85–9.64)	10.70 (8.68–13.17)	14.87 (12.46–17.73)	—	—	—	---
Model 1	—	Ref.	1.40 (1.01–1.93)	1.76 (1.28–2.41)	1.32 (1.13–1.54)	0.0004	1.33 (1.17–1.51)	<.0001
Model 2	—	Ref.	1.35 (0.97–1.88)	1.60 (1.12–2.29)	1.26 (1.05–1.50)	0.0112	1.34 (1.14–1.57)	0.0005

The primary outcome was the occurrence of stroke, acute coronary syndrome, acute decompensated heart failure, atrial fibrillation, coronary revascularization, and all-cause mortality

Model 1 adjusted for the treatment group, sex, age, and study site. Model 2 adjusted for the treatment group, sex, age, BMI, study site, evening home systolic blood pressure at baseline, heart rate, glucose level, coronary heart disease history, smoking, and alcohol use. Ref. indicates reference; and *P*_trend_, *P* value for trend. *IR indicates incidence rate, per 1000 person-years

**Fig. 2:**
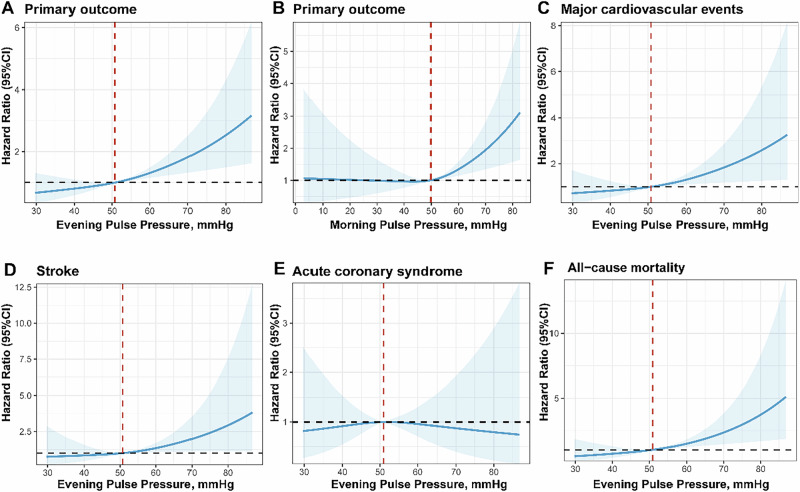
Restricted cubic spline curve between home pulse pressure and outcomes. The association between home pulse pressure and outcomes was evaluated using restricted cubic splines. The analyses were adjusted for baseline characteristics, including treatment group, sex, age, study site, BMI, evening home systolic blood pressure, heart rate, glucose level, coronary heart disease history, smoking, and alcohol use. Shaded areas indicate 95% CIs

As a continuous variable, evening PP was consistently positively associated with composite events in model 1 (HR = 1.33, 95% CI: 1.17–1.51, *P* < 0.0001) and model 2 (HR = 1.34, 95% CI: 1.14–1.57, *P* = 0.0005). For each 10 mmHg increase in evening PP, the risk of outcome events increased by ~33% in both models. To better explain the observed association, we further analyzed evening PP and morning PP using cubic spline regression in the final adjusted model. Results of cubic spline regression are displayed in Fig. 2, where the evening home PP value of 50 mmHg was chosen as a reference.

In our further analysis, each 10 mmHg increase in evening PP was significantly associated with increased risk of MACE and all-cause mortality in models 1 and 2. However, when analyzing PP as a categorical variable, MACE only showed a significant positive association in model 1 (Table 3).

**Table 3:** Adjusted association of evening home pulse pressure groups and secondary outcome

Outcome	Overall (*n* = 7703)	Tertile 1(*n* = 2568)	Tertile 2 (*n* = 2568)	Tertile 3(*n* = 2567)	Per one stage increase		Per 10 mmHg increase	
	Outcomes	HR (95% CI)	HR (95% CI)	HR (95% CI)	HR (95% CI)	*P*_trend_	HR (95% CI)	*P*_value_
Stroke								
Model 1	71	Ref.	1.32 (0.68–2.56)	1.94 (1.04–3.63)	1.40 (1.03–1.91)	0.0314	1.45(11.14–1.85)	0.0028
Model 2			1.17 (0.60–2.29)	1.34 (0.66–2.72)	1.16 (0.81–1.64)	0.4214	1.25 (0.91–1.71)	0.1623
Acute coronary syndrome								
Model 1	87	Ref.	1.27 (0.74–2.15)	1.10 (0.63–1.92)	1.04 (0.80–1.37)	0.7570	0.97 (0.76–1.24)	0.7939
Model 2			1.29 (0.74–2.24)	1.21 (0.63–2.30)	1.10 (0.80–1.51)	0.5650	1.02(0.74–1.38)	0.9250
Major cardiovascular events								
Model 1	161	Ref.	1.36 (0.89–2.08)	1.63 (1.08–2.47)	1.27 (1.04–1.56)	0.0209	1.29(1.08–1.53)	0.0039
Model 2			1.27 (0.82–1.97)	1.42 (0.88–2.28)	1.18 (0.94–1.50)	0.1583	1.26 (1.01–1.56)	0.0371
All-cause mortality								
Model 1	92	Ref.	1.62 (0.85–3.07)	2.68 (1.47–4.89)	1.64 (1.23–2.18)	0.0006	1.53 (1.24–1.89)	<0.0001
Model 2			1.67 (0.87–3.21)	2.82 (1.45–5.48)	1.68 (1.22–2.31)	0.0015	1.71 (1.30–2.24)	0.0001

Model 1 adjusted for the treatment group, sex, age, and study site. Model 2 adjusted for the treatment group, sex, age, BMI, study site, evening home systolic blood pressure at baseline, heart rate, glucose level, coronary heart disease history, smoking, and alcohol use. Ref. indicates reference; HR, hazard ratio; and *P*_trend_, *P* value for trend

### Sensitivity analyses

We performed sensitivity analyses on the association between evening home PP and composite events to address the possibility of reverse causality, and all results remained consistent in the multivariable Cox regression analysis (Supplementary Tables 1). In the models, the association between PP and composite events was even higher after excluding participants with a coronary heart disease history.

For morning SBP and PP as categorical variables, no significant association was observed in either model 1 or model 2, suggesting the association between the primary outcome and morning SBP, as well as morning PP, did not reach the same level of significance observed for evening PP (Supplementary Tables 2, 3 and 5). For evening SBP, no significant association was observed between the second tertile of evening SBP and outcomes compared to the first tertile (Supplementary Tables 4 and 5). For each 10 mmHg increase in morning SBP, PP and evening SBP, both models showed lower risk of outcome events compared to evening PP (Supplementary Table 5).

To test whether morning home PP, along with evening home SBP, improved the model prediction performance for outcome events, we calculated four NRI indicators. In the traditional model, we controlled for the treatment group, sex, age, BMI, studyevening/evening SBP, heart rate, glucose level, coronary heart disease history, smoking, and alcohol use. Adding evening PP improved prognostic performance (NRI: 0.16, 95% CI: 0.02–0.29, *P* = 0.0229), whereas neither morning SBP, PP, nor evening SBP showed similar improvement (Table 4). These findings suggest that PP alone provides added prognostic information for the prediction of outcome events.

**Table 4:** NRI for incremental prognostic value assessment

Model	NRI Estimate (95% CI), %	*P* value
Traditional Model^a^	Ref.	—
Traditional Model + Morning SBP	8.57 (−2.94–23.75)	0.2093
Traditional Model + Morning PP	11.10 (−2.37–25.73)	0.1342
Traditional Model + Evening SBP	6.00 (−6.27–20.43)	0.3791
Traditional Model + Evening PP	15.90 (2.19–29.46)	0.0229

^a^The Traditional Model was controlled for the treatment group, sex, age, BMI, study site, morning/evening systolic blood pressure at baseline, heart rate, glucose level, coronary heart disease history, smoking, and alcohol use. Ref. indicates reference

## Discussion

The present research examined, for the first time, the prognostic values of evening home-measured PP in predicting CVD risk and mortality among Chinese older adults with hypertension using a large sample from a multicenter RCT trial. Evening PP showed better performance in predicting composite events after adjusting for all potential confounders. Subsequent sensitivity analysis excluded reverse causality, further confirming the robustness of the association between evening home PP and composite events. When measuring BP, it is important to consider evening home PP as it can provide enhanced personalized assessment and improve CVD risk prediction for older adults with hypertension.

We have discovered that evening PP is a stronger predictive marker of outcome events than morning SBP, PP, and evening SBP in an older adult population with hypertension, using a simple bedtime BP measurement. The result remained consistent after controlling for some well-known predictors of CVD, suggesting that PP provided added prognostic information and could be potentially used as a unique and robust predictor for CVD risk assessment. This finding is generally in agreement with previous studies. In a cross-sectional study among patients with diabetes mellitus, both morning and evening PP were positively associated with CVD events, with morning PP exhibiting a stronger association [8]. Notably, the association between evening PP and CVD risk was more remarkable in individuals aged over 60 years [8]. Interestingly, our study showed that morning PP was significantly and positively associated with CVD risk when treated as a continuous variable. However, such an association disappeared when morning PP was treated as a categorical variable, indicating that a categorical analysis might lose statistical impact compared to a continuous analysis. In general, our results suggested that evening home PP provided a more consistent and robust prediction ability for CVD risk assessment, regardless of the statistical method used. Therefore, evening home PP holds promise for assisting individuals with hypertension, especially older adults, in monitoring their CVD health more efficiently and sustainably.

Noteworthily, our study showed that evening home PP predicted the risk of stroke but not coronary artery disease (CAD), despite the well-demonstrated positive association between PP and CAD in the broad literature [25–28]. This finding seemed to contradict a large number of studies showing CAD as the most commonly reported adverse health outcome related to PP [25–28]. Although unexpected, our result was consistent with Hoshide et al.’s study in a Japanese population, which showed that both morning and evening home PP were associated with stroke risk, while neither morning nor evening home PP was associated with CAD risk [12]. One probable explanation may be that Asian countries have a higher burden of stroke, while Western countries have a higher burden of CAD [29, 30]. Our study was conducted in an Asian population with more prevalent stroke cases that were more sensitive to BP, making it easier to observe a significant association between PP and stroke risk [12, 31]. On the other hand, we did not observe a significant effect of PP on CAD risk, which might be due to the confounding effects of antihypertensive medication use in our study, as this was an intervention study with BP control. A previous study observed a significant positive association between home SBP and CAD risk among untreated individuals. However, such an association disappeared in patients on antihypertensive medication, suggesting treatment might be a potential confounder [32]. In addition, our study showed a significant positive association between evening home PP and all-cause mortality, which might be explained mainly by the high mortality risk related to stroke.

Currently, there are no standard guidelines specifying the target ranges of PP. Still, high PP is an independent risk factor for CVD events and target organ damage, independent of SBP and mean BP [33, 34]. It is noteworthy that in older adults with hypertension, PP may be a superior predictor of CVD risk than SBP and DBP [35]. Several studies attempted to establish hypertension diagnostic and CVD prognosis cut-offs derived from HBPM, yet without convincing conclusions [36, 37]. To our knowledge, only an earlier study suggested that a home PP of ≥76 mmHg predicted all CVD outcomes except stroke in the older population. They included 6470 participants from the IDHOCO. Still, the HBPM was not standardized in terms of device types, number of measurements, and intervals between readings [38]. Our study had a large sample size, and we provided clear instructions on the use of home BP monitors to improve the reliability of BP measurement. In this study, we have found that when the evening home PP exceeds 50 mmHg, the composite events risk increases with rising PP. Consequently, it is crucial to determine a safe and optimal home PP threshold for individuals with hypertension.

## Implications

Our study findings suggested that it was important to consider not only the SBP and DBP ranges but also the PP range, thus carrying significant implications in clinical practice. PP should be included as an important prognostic factor for CVD in future guidelines to help identify high-risk populations and take proactive interventions. In addition, our findings showed that HBPM was a reliable and cost-effective approach for BP monitoring and had the potential to be widely used for active self-monitoring and control of BP at home. Using a validated device, following standard procedures, and providing patient education and training were essential to ensure reliable and accurate BP assessment. Finally, further research is warranted to establish appropriate PP cut-off values for assessing CVD risk, thereby facilitating the establishment of a scientifically grounded PP threshold in clinical guidelines.

### Limitations

Based on the well-organized data from the STEP trial, we utilized home BP data collected within one year of enrollment rather than relying solely on baseline data. This approach bolstered the reliability of our findings. Nonetheless, it is imperative to acknowledge certain limitations inherent in our study. First, it should be noted that the STEP trial specifically enrolled Chinese hypertensive adults, thereby the data to access the association between PP measurements and adverse health outcomes in normotensive populations was limited, which would be the direction of future studies. Second, the statistical validity of this study may have been compromised by suboptimal adherence to HBPM protocols and the absence of home BP data for over 800 participants. It is suggested that more education and training should be provided to older adults with hypertension to improve adherence. Third, our study measured evening home PP during the period of 6:00p.m. to 9:00 p.m. This evening time measurement was similar to some European studies but different from the J-HOP Study, Ohasama Study, and HOMED-BP study in Japan, which measured BP before going to bed [12, 13, 39]. The inconsistent evening PP measurement periods might have an impact on the results due to the different evening activities, such as dinner time and frequency, exercise, bathing, and alcohol use. The discrepancy in evening measurement times used in various studies indicates the need for a more consistent time definition to facilitate cross-study comparisons. Additionally, adding 24-h ambulatory BP measurements might be necessary to provide a more comprehensive and detailed analysis of the timing effect of PP measurement. Finally, this is a secondary analysis of data from a randomized controlled trial; although we fully considered the reverse causality, further primary studies are warranted to confirm our findings.

### Perspective of Asia

Current evidence suggests that the predictive value of evening home pulse pressure for cardiovascular events and mortality varies across different populations, which may be attributed to different disease burdens between Asian and Western countries [29, 30]. Future studies should validate its applicability across diverse populations to facilitate clinical translation.

## Conclusions

In summary, our study showed that elevated evening home PP was associated with a higher risk of composite events. These findings underscore the potential of evening home PP as a valuable predictive indicator in CVD prognosis and mortality.

## Supplementary information

Supplemental Material

## Data Availability

The data supporting the findings of this study are available from the corresponding author upon reasonable request.

## References

[1] Zhou B, Perel P, Mensah GA, Ezzati M. Global epidemiology, health burden and effective interventions for elevated blood pressure and hypertension. Nat Rev Cardiol. 2021;18:785–802. 34050340 10.1038/s41569-021-00559-8 PMC8162166

[2] Fuchs FD, Whelton PK. High blood pressure and cardiovascular disease. Hypertens Dallas Tex. 2020;75:285–92. 10.1161/HYPERTENSIONAHA.119.14240 PMC10243231 31865786

[3] Zhang W-L, Cai J. STEP to blood pressure management of elderly hypertension: evidence from Asia. Hypertens Res J Jpn Soc Hypertens. 2022;45:576–82. 10.1038/s41440-022-00875-7 PMC8923999 35277670

[4] Liu J, Liu X, Liu G, Hu B. Research on the impact of mutual elderly care on the physical and mental health of rural elderly--an empirical study based on the propensity score matching method (PSM). 2024. Available from: https://www.researchsquare.com/article/rs-4898207/v1.

[5] Divisón-Garrote JA, Velilla-Zancada S, Artigao-Rodenas LM, García-Lerín A, Vicente-Molinero A, Piera Carbonell AM, et al. Home blood pressure self-measurement: “current situation and new perspectives. Hipertens Riesgo Vasc. 2023;40:85–97. 36114104 10.1016/j.hipert.2022.07.005

[6] Tomitani N, Hoshide S, Kario K. The importance of regular home blood pressure monitoring over the life course. Hypertens Res J Jpn Soc Hypertens. 2024;47:540–2. 10.1038/s41440-023-01492-8 37891337

[7] Kario K. Home blood pressure monitoring: current status and new developments. Am J Hypertens. 2021;34:783–94. 34431500 10.1093/ajh/hpab017 PMC8385573

[8] Takegami M, Ushigome E, Hata S, Yoshimura T, Kitagawa N, Hasegawa G, et al. Home-measured pulse pressure is a predictor of cardiovascular disease in type 2 diabetes: the KAMOGAWA-HBP study. Nutr Metab Cardiovasc Dis NMCD. 2022;32:2330–7. 36100493 10.1016/j.numecd.2022.08.006

[9] Shimbo D, Artinian NT, Basile JN, Krakoff LR, Margolis KL, Rakotz MK, et al. Self-measured blood pressure monitoring at home: a joint policy statement from the American Heart Association and American Medical Association. Circulation. 2020;142:e42–63. 32567342 10.1161/CIR.0000000000000803

[10] Cheng H-M, Lin H-J, Wang T-D, Chen C-H. Asian management of hypertension: current status, home blood pressure, and specific concerns in Taiwan. J Clin Hypertens Greenwich Conn. 2020;22:511–4. 10.1111/jch.13747 PMC8030078 31816161

[11] Mancia G, Kreutz R, Brunström M, Burnier M, Grassi G, Januszewicz A, et al. 2023 ESH Guidelines for the management of arterial hypertension The Task Force for the management of arterial hypertension of the European Society of Hypertension: endorsed by the International Society of Hypertension (ISH) and the European Renal Association (ERA). J Hypertens. 2023;41:1874–2071. 37345492 10.1097/HJH.0000000000003480

[12] Hoshide S, Yano Y, Haimoto H, Yamagiwa K, Uchiba K, Nagasaka S, et al. Morning and evening home blood pressure and risks of incident stroke and coronary artery disease in the Japanese general practice population: the Japan Morning Surge-Home Blood Pressure Study. Hypertens Dallas Tex. 2016;68:54–61. 10.1161/HYPERTENSIONAHA.116.07201 27160200

[13] Asayama K, Ohkubo T, Kikuya M, Obara T, Metoki H, Inoue R, et al. Prediction of stroke by home “morning” versus “evening” blood pressure values: the Ohasama study. Hypertens Dallas Tex. 2006;48:737–43. 10.1161/01.HYP.0000240332.01877.11 16952977

[14] Niiranen TJ, Johansson JK, Reunanen A, Jula AM. Optimal schedule for home blood pressure measurement based on prognostic data: the Finn-Home Study. Hypertens Dallas Tex. 2011;57:1081–6. 10.1161/HYPERTENSIONAHA.110.162123 21482956

[15] Asayama K, Ohkubo T, Hara A, Hirose T, Yasui D, Obara T, et al. Repeated evening home blood pressure measurement improves prognostic significance for stroke: a 12-year follow-up of the Ohasama study. Blood Press Monit. 2009;14:93–8. 19359986 10.1097/MBP.0b013e32832a9d91

[16] Umemura S, Arima H, Arima S, Asayama K, Dohi Y, Hirooka Y, et al. The Japanese society of hypertension guidelines for the management of hypertension (JSH 2019). Hypertens Res J Jpn Soc Hypertens. 2019;42:1235–481. 10.1038/s41440-019-0284-9 31375757

[17] Makkiyah FA, Dicha S, Nurrizzka RH. A single-center experience of correlation of pulse pressure to mortality of stroke hemorrhage patients in Indonesia. ScientificWorldJournal. 2023;2023:5517493. 37593547 10.1155/2023/5517493 PMC10432090

[18] Huang J, Liu L, Huang Y-Q, Lo K, Yu Y-L, Chen C-L, et al. Association between pulse pressure and ischaemic stroke in elderly patients with hypertension. Postgrad Med J. 2021;97:222–6. 32300056 10.1136/postgradmedj-2019-137357

[19] Khanna AK, Kinoshita T, Natarajan A, Schwager E, Linn DD, Dong J, et al. Association of systolic, diastolic, mean, and pulse pressure with morbidity and mortality in septic ICU patients: a nationwide observational study. Ann Intensive Care. 2023;13:9. 36807233 10.1186/s13613-023-01101-4 PMC9941378

[20] Zhang W, Zhang S, Deng Y, Wu S, Ren J, Sun G, et al. Trial of intensive blood-pressure control in older patients with hypertension. N Engl J Med. 2021;385:1268–79. 34491661 10.1056/NEJMoa2111437

[21] Zhang S, Wu S, Ren J, Chen X, Zhang X, Feng Y, et al. Strategy of blood pressure intervention in the elderly hypertensive patients (STEP): rational, design, and baseline characteristics for the main trial. Contemp Clin Trials. 2020;89:105913. 31838255 10.1016/j.cct.2019.105913

[22] Meng L, Zhao D, Pan Y, Ding W, Wei Q, Li H, et al. Validation of Omron HBP-1300 professional blood pressure monitor based on auscultation in children and adults. BMC Cardiovasc Disord. 2016;16:9. 26758197 10.1186/s12872-015-0177-z PMC4711064

[23] Kim JH. Multicollinearity and misleading statistical results. Korean J Anesthesiol. 2019;72:558–69. 31304696 10.4097/kja.19087 PMC6900425

[24] Leening MJG, Vedder MM, Witteman JCM, Pencina MJ, Steyerberg EW. Net reclassification improvement: computation, interpretation, and controversies: a literature review and clinician’s guide. Ann Intern Med. 2014;160:122–31. 24592497 10.7326/M13-1522

[25] Franklin SS, Lopez VA, Wong ND, Mitchell GF, Larson MG, Vasan RS, et al. Single versus combined blood pressure components and risk for cardiovascular disease: the Framingham Heart Study. Circulation. 2009;119:243–50. 19118251 10.1161/CIRCULATIONAHA.108.797936 PMC3042701

[26] Franklin SS, Khan SA, Wong ND, Larson MG, Levy D. Is pulse pressure useful in predicting risk for coronary heart Disease? The Framingham Heart Study. Circulation. 1999;100:354–60. 10421594 10.1161/01.cir.100.4.354

[27] Assmann G, Cullen P, Evers T, Petzinna D, Schulte H. Importance of arterial pulse pressure as a predictor of coronary heart disease risk in PROCAM. Eur Heart J. 2005;26:2120–6. 16141262 10.1093/eurheartj/ehi467

[28] Said MA, Eppinga RN, Lipsic E, Verweij N, van der Harst P. Relationship of arterial stiffness index and pulse pressure with cardiovascular disease and mortality. J Am Heart Assoc. 2018;7:e007621. 29358193 10.1161/JAHA.117.007621 PMC5850166

[29] Kim AS, Johnston SC. Global variation in the relative burden of stroke and ischemic heart disease. Circulation. 2011;124:314–23. 21730306 10.1161/CIRCULATIONAHA.111.018820

[30] Ueshima H, Sekikawa A, Miura K, Turin TC, Takashima N, Kita Y, et al. Cardiovascular disease and risk factors in Asia: a selected review. Circulation. 2008;118:2702–9. 19106393 10.1161/CIRCULATIONAHA.108.790048 PMC3096564

[31] Perkovic V, Huxley R, Wu Y, Prabhakaran D, MacMahon S. The burden of blood pressure-related disease: a neglected priority for global health. Hypertens Dallas Tex. 2007;50:991–7. 10.1161/HYPERTENSIONAHA.107.095497 17954719

[32] Niiranen TJ, Asayama K, Thijs L, Johansson JK, Ohkubo T, Kikuya M, et al. Outcome-driven thresholds for home blood pressure measurement: international database of home blood pressure in relation to cardiovascular outcome. Hypertens Dallas Tex. 2013;61:27–34. 10.1161/HYPERTENSIONAHA.111.00100 PMC3607331 23129700

[33] Mancusi C, Losi MA, Izzo R, Canciello G, Carlino MV, Albano G, et al. Higher pulse pressure and risk for cardiovascular events in patients with essential hypertension: the Campania Salute Network. Eur J Prev Cardiol. 2018;25:235–43. 29226693 10.1177/2047487317747498

[34] Blacher J, Staessen JA, Girerd X, Gasowski J, Thijs L, Liu L, et al. Pulse pressure not mean pressure determines cardiovascular risk in older hypertensive patients. Arch Intern Med. 2000;160:1085–9. 10789600 10.1001/archinte.160.8.1085

[35] Tsai T-Y, Cheng H-M, Chuang S-Y, Chia Y-C, Soenarta AA, Minh HV, et al. Isolated systolic hypertension in Asia. J Clin Hypertens Greenwich Conn. 2021;23:467–74. 10.1111/jch.14111 PMC8029528 33249701

[36] Kario K, Okawara Y, Kanegae H, Hoshide S. Potential long-term benefit of home systolic blood pressure below 125 mm Hg for cardiovascular risk reduction: the J-HOP study extended. Hypertens Dallas Tex. 2024;81:282–90. 10.1161/HYPERTENSIONAHA.123.22122 38073531

[37] Bryant KB, Green MB, Shimbo D, Schwartz JE, Kronish IM, Zhang Y, et al. Home blood pressure monitoring for hypertension diagnosis by current recommendations: a long way to go. Hypertens Dallas Tex. 2022;79:e15–7. 10.1161/HYPERTENSIONAHA.121.18463 PMC8754001 34852639

[38] Aparicio LS, Thijs L, Asayama K, Barochiner J, Boggia J, Gu Y-M, et al. Reference frame for home pulse pressure based on cardiovascular risk in 6470 subjects from 5 populations. Hypertens Res J Jpn Soc Hypertens. 2014;37:672–8. 10.1038/hr.2014.45 24646650

[39] Watabe D, Asayama K, Hanazawa T, Hosaka M, Satoh M, Yasui D, et al. Predictive power of home blood pressure indices at baseline and during follow-up in hypertensive patients: HOMED-BP study. Hypertens Res J Jpn Soc Hypertens. 2018;41:622–8. 10.1038/s41440-018-0050-4 29808033

