# Supplementary material for: Evening home pulse pressure predicted cardiovascular events and mortality in older adults with hypertension: findings based on the STEP trial
Source: Hypertens Res. 2025 Aug 26;48(11):2801–10. doi: 10.1038/s41440-025-02349-y (PMC12586171; doi:10.1038/s41440-025-02349-y)
Supplement: Supplementary file 1 — Supplemental Material [file 41440_2025_2349_MOESM1_ESM.docx]

Supplemental Material

**Supplementary Figure 1 Participant selection flowchart**


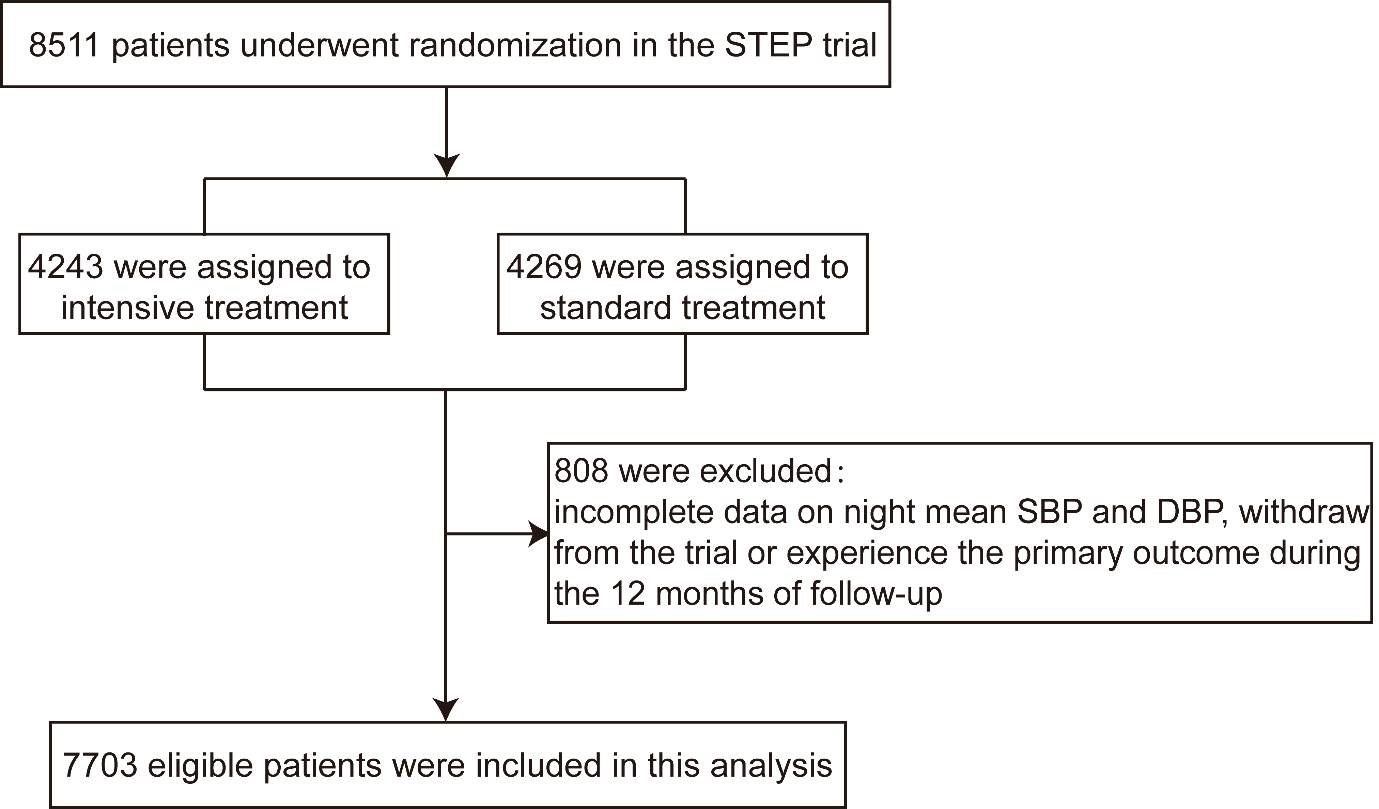


**Supplementary Table 1. Sensitivity analysis excluding participants with a history of coronary heart disease, according to the evening home pulse pressure (subgroups or per 10 mmHg increase)**

|  | **Overall** | **Tertile 1**  (N=2568) | **Tertile 2**  (N=2568) | **Tertile 3**  (N=2567) | **Per one stage increase** | ***P* _trend_** | **Per 10 mmHg increase** | ***P* _value_** |
| --- | --- | --- | --- | --- | --- | --- | --- | --- |
| **Excluding participants with a coronary heart disease history** | | | | | | | | |
| Participants(n) | 7250 | 2447 | 2403 | 2440 | - | - | - | **-** |
| Outcomes(n) | 256 | 59 | 83 | 114 | - | - | - | **-** |
| IR*(95% CI) | 10.54  (9.31-11.93) | 7.16  (5.50-9.29) | 10.30  (8.26-12.82) | 14.28  (11.84-17.19) | - | - | - | **-** |
| Model 1 | - | Ref. | 1.43  (1.02-2.01) | 1.81  (1.30-2.53) | 1.34  (1.14-1.57) | 0.0004 | 1.35  (1.18-1.54) | < .0001 |
| Model 2 | - | Ref. | 1.41  (1.00-2.00) | 1.71  (1.17-2.50) | 1.30  (1.08-1.56) | 0.0056 | 1.37  (1.16-1.62) | 0.0002 |

Model 1 adjusted for the treatment group, sex, age, and study site. Model 2 adjusted for the treatment group, sex, age, BMI, study site, evening home systolic blood pressure at baseline, heart rate, glucose level, smoking, and alcohol use. Ref. indicates reference; and *P* _trend_, P value for trend.

*IR, indicates incidence rate, per 1000 person-years.

**Supplementary Table 2. Baseline participant characteristics across morning home systolic blood pressure tertiles**

| **Variables** | **Tertile 1**  (N=2567) | **Tertile 2**  (N=2569) | **Tertile 3**  (N=2567) |
| --- | --- | --- | --- |
| Age, y | 65.62 (4.66) | 66.18 (4.73) | 66.57 (4.89) |
| Male, n (%) | 1080 (42.1) | 1220 (47.5) | 1294 (50.4) |
| BMI, kg/m^2^ | 25.14 (3.02) | 25.72 (3.14) | 25.90 (3.25) |
| Morning SBP during follow-up, mmHg | 121.44 (4.24) | 130.07 (2.01) | 139.89 (5.76) |
| Home blood pressure at baseline | | | |
| Morning SBP, mmHg | 120.52 (7.32) | 129.54 (6.97) | 139.19 (9.27) |
| Morning DBP, mmHg | 77.08 (7.28) | 80.21 (7.56) | 83.48 (8.52) |
| Morning PP, mmHg | 43.44 (7.65) | 49.33 (8.04) | 55.71 (9.75) |
| Morning HR, bpm | 73.81 (10.18) | 73.36 (10.29) | 73.52 (10.54) |
| Smoking, n (%) | | | |
| Currently | 302 (11.8) | 432 (16.8) | 517 (20.1) |
| Never | 1956 (76.2) | 1833 (71.4) | 1731 (67.4) |
| Former | 309 (12.0) | 304 (11.8) | 319 (12.4) |
| Alcohol use, n (%) | | | |
| Currently | 586 (22.8) | 682 (26.5) | 765 (29.8) |
| Never | 1858 (72.4) | 1765 (68.7) | 1655 (64.5) |
| Former | 123 (4.8) | 122 (4.7) | 147 (5.7) |
| Lipid profile | | | |
| Triglyceride, mmol/L | 1.52 (0.96) | 1.61 (1.03) | 1.66 (1.20) |
| TC, mmol/L | 4.83 (1.08) | 4.90 (1.11) | 4.95 (1.08) |
| HDL-C, mmol/L | 1.27 (0.31) | 1.26 (0.30) | 1.25 (0.31) |
| LDL-C, mmol/L | 2.65 (0.88) | 2.70 (0.88) | 2.74 (0.88) |
| FBG, mmol/L | 6.00 (1.40) | 6.13 (1.54) | 6.27 (1.71) |
| Creatinine, μmol/L | 71.84 (16.85) | 73.35 (18.44) | 74.11 (18.28) |
| With DM history, n (%) | 446 (17.4) | 483 (18.8) | 541 (21.1) |
| With CHD history, n (%) | 156 (6.1) | 159 (6.2) | 138 (5.4) |

Values are presented as the mean ± SD or n (%), as appropriate. BMI, body mass index, SBP, systolic blood pressure; DBP, diastolic blood pressure; HR, heart rate; TC, total cholesterol; HDL-C, high-density lipoprotein cholesterol; LDL-C, low-density lipoprotein cholesterol; FBG, fasting blood glucose; DM, diabetes mellitus; CHD, coronary heart disease.

**Supplementary Table 3. Baseline participant characteristics across morning home pulse pressure tertiles**

| **Variables** | **Tertile 1**  (N=2573) | **Tertile 2**  (N=2565) | **Tertile 3**  (N=2565) |
| --- | --- | --- | --- |
| Age, y | 64.57 (4.11) | 66.03 (4.63) | 67.76 (5.01) |
| Male, n (%) | 1361 (52.9) | 1178 (45.9) | 1055 (41.1) |
| BMI, kg/m^2^ | 25.54 (3.03) | 25.58 (3.10) | 25.64 (3.32) |
| Morning PP during follow-up, mmHg | 40.67 (5.45) | 49.79 (2.04) | 59.87 (5.86) |
| Home blood pressure at baseline | | | |
| Morning SBP, mmHg | 123.82 (9.07) | 129.50 (9.51) | 135.94 (10.77) |
| Morning DBP, mmHg | 83.21 (7.21) | 80.55 (7.98) | 76.99 (8.25) |
| Morning PP, mmHg | 40.61 (5.54) | 48.95 (5.52) | 58.95 (7.97) |
| Morning HR, bpm | 74.98 (10.02) | 73.88 (10.65) | 71.83 (10.10) |
| Smoking, n (%) | | | |
| Currently | 456 (17.7) | 421 (16.4) | 374 (14.6) |
| Never | 1750 (68.0) | 1868 (72.8) | 1902 (74.2) |
| Former | 367 (14.3) | 276 (10.8) | 289 (11.3) |
| Alcohol use, n (%) | | | |
| Currently | 781 (30.4) | 661 (25.8) | 591 (23.0) |
| Never | 1653 (64.2) | 1782 (69.5) | 1843 (71.9) |
| Former | 139 (5.4) | 122 (4.8) | 131 (5.1) |
| Lipid profile | | | |
| Triglyceride, mmol/L | 1.58 (1.00) | 1.59 (1.08) | 1.62 (1.13) |
| TC, mmol/L | 4.87 (1.08) | 4.87 (1.10) | 4.93 (1.09) |
| HDL-C, mmol/L | 1.26 (0.31) | 1.26 (0.30) | 1.26 (0.31) |
| LDL-C, mmol/L | 2.68 (0.87) | 2.68 (0.89) | 2.72 (0.88) |
| FBG, mmol/L | 5.91 (1.40) | 6.15 (1.57) | 6.33 (1.66) |
| Creatinine, μmol/L | 74.19 (17.51) | 72.42 (17.48) | 72.70 (18.61) |
| With DM history, n (%) | 324 (12.6) | 491 (19.1) | 655 (25.5) |
| With CHD history, n (%) | 127 (4.9) | 169 (6.6) | 157 (6.1) |

Values are presented as the mean ± SD or n (%), as appropriate. BMI, body mass index, SBP, systolic blood pressure; DBP, diastolic blood pressure; HR, heart rate; TC, total cholesterol; HDL-C, high-density lipoprotein cholesterol; LDL-C, low-density lipoprotein cholesterol; FBG, fasting blood glucose; DM, diabetes mellitus; CHD, coronary heart disease.

**Supplementary Table 4. Baseline participant characteristics across evening home systolic blood pressure tertiles**

| **Variables** | **Tertile 1**  (N=2567) | **Tertile 2**  (N=2568) | **Tertile 3**  (N=2568) |
| --- | --- | --- | --- |
| Age, y | 65.56 (4.57) | 66.22 (4.84) | 66.57 (4.86) |
| Male, n (%) | 1095 (42.7) | 1185 (46.1) | 1314 (51.2) |
| BMI, kg/m^2^ | 25.13 (3.02) | 25.68 (3.10) | 25.94 (3.28) |
| Evening SBP during follow-up, mmHg | 121.04 (4.39) | 129.90 (2.02) | 140.07 (5.93) |
| Home blood pressure at baseline | | | |
| Evening SBP, mmHg | 120.18 (7.51) | 129.00 (7.04) | 139.13 (10.00) |
| Evening DBP, mmHg | 75.77 (7.04) | 78.64 (7.34) | 81.34 (8.35) |
| Evening PP, mmHg | 44.41 (7.58) | 50.36 (7.78) | 57.79 (9.95) |
| Evening HR, bpm | 73.65 (10.29) | 73.60 (10.25) | 73.45 (10.48) |
| Smoking, n (%) | | | |
| Currently | 288 (11.2) | 383 (14.9) | 580 (22.6) |
| Never | 1965 (76.5) | 1885 (73.4) | 1670 (65.0) |
| Former | 314 (12.2) | 300 (11.7) | 318 (12.4) |
| Alcohol use, n (%) | | | |
| Currently | 635 (24.7) | 664 (25.9) | 734 (28.6) |
| Never | 1809 (70.5) | 1792 (69.8) | 1677 (65.3) |
| Former | 123 (4.8) | 112 (4.4) | 157 (6.1) |
| Lipid profile | | | |
| Triglyceride, mmol/L | 1.52 (1.02) | 1.62 (1.07) | 1.65 (1.12) |
| TC, mmol/L | 4.85 (1.10) | 4.91 (1.11) | 4.91 (1.07) |
| HDL-C, mmol/L | 1.28 (0.31) | 1.26 (0.31) | 1.24 (0.30) |
| LDL-C, mmol/L | 2.66 (0.87) | 2.70 (0.89) | 2.72 (0.87) |
| FBG, mmol/L | 5.94 (1.36) | 6.13 (1.55) | 6.32 (1.72) |
| Creatinine, μmol/L | 71.92 (17.00) | 73.30 (18.04) | 74.08 (18.54) |
| With DM history, n (%) | 401 (15.6) | 490 (19.1) | 579 (22.5) |
| With CHD history, n (%) | 162 (6.3) | 151 (5.9) | 140 (5.5) |

Values are presented as the mean ± SD or n (%), as appropriate. BMI, body mass index, SBP, systolic blood pressure; DBP, diastolic blood pressure; HR, heart rate; TC, total cholesterol; HDL-C, high-density lipoprotein cholesterol; LDL-C, low-density lipoprotein cholesterol; FBG, fasting blood glucose; DM, diabetes mellitus; CHD, coronary heart disease.

**Supplementary Table 5. Association of morning home systolic blood pressure, pulse pressure and evening home systolic blood pressure (per 10 mmHg increase or by subgroup) with primary outcome**

|  | **Overall** | **Tertile 1** | **Tertile 2** | **Tertile 3** | **Per one stage increase** | ***P* _trend_** | **Per 10 mmHg increase** | ***P* _value_** |
| --- | --- | --- | --- | --- | --- | --- | --- | --- |
| **Morning home systolic blood pressure** | | | | | | | | |
| N | 7703 | 2567 | 2560 | 2567 | - | - | - | - |
| Outcomes(n) | 284 | 87 | 95 | 102 | - | - | - | - |
| IR*(95% CI) |  | 10.10  (8.14-12.5) | 11.04  (8.99-13.54) | 11.92  (9.78,14.51) | - | - | - | - |
| Model 1 | - | Ref. | 1.10  (0.82-1.48) | 1.23  (0.91-1.66) | 1.11  (0.95-1.29) | 0.1790 | 1.18  (1.03-1.36) | 0.0178 |
| Model 2 | - | Ref. | 1.00  (0.74-1.36) | 1.02  (0.72-1.44) | 1.01  (0.85-1.20) | 0.9173 | 1.11  (0.94-1.31） | 0.2268 |
| **Morning home pulse pressure** | | | | | | | | |
| N | 7703 | 2573 | 2565 | 2565 | - | - | - | - |
| Outcomes(n) | 284 | 79 | 90 | 115 | - | - | - | - |
| IR*(95% CI) | 11.02  (9.79-12.39) | 9.13  (7.28-11.42) | 10.49  (8.49-12.94) | 13.45  (11.17-16.19) | - | - | - | - |
| Model 1 | - | Ref. | 1.14  (0.83-1.55) | 1.31  (0.96-1.78) | 1.14  (0.98-1.33) | 0.0852 | 1.25  (1.09-1.43) | 0.0015 |
| Model 2 | - | Ref. | 1.05  (0.76-1.44) | 1.16  (0.82-1.65) | 1.08  (0.91-1.29) | 0.3845 | 1.23  (1.04-1.45) | 0.0140 |
| **Evening home systolic blood pressure** | | | | | | | | |
| N | 7703 | 2567 | 2568 | 2568 | - | - | - | - |
| Outcomes(n) | 284 | 81 | 78 | 125 | - | - | - | - |
| IR*(95% CI) | 7703 | 9.39  (7.51,11.72) | 9.04  (7.2,11.33) | 14.65  (12.26,17.49) | - | - | - | - |
| Model 1 | - | Ref. | 0.94  (0.68-1.28) | 1.64  (1.22-2.19) | 1.31  (1.12-1.52) | 0.0006 | 1.30  (1.14-1.48) | <.0001 |
| Model 2 | - | Ref. | 0.92  (0.66-1.30) | 1.60  (1.07-2.38) | 1.29  (1.05-1.58) | 0.0161 | 1.29  (1.11-1.52) | 0.0014 |

Model 1 adjusted for the treatment group, sex, age, and study site. Model 2 adjusted for the treatment group, sex, age, BMI, study site, morning/evening home systolic blood pressure at baseline, heart rate, glucose level, coronary heart disease history, smoking, and alcohol use. Ref. indicates reference; and *P* _trend_, P value for trend.

*IR, indicates incidence rate, per 1000 person-years.
